# Keeping up appearances: Strategic information exchange by disidentified group members

**DOI:** 10.1371/journal.pone.0175155

**Published:** 2017-04-06

**Authors:** Jort de Vreeze, Christina Matschke

**Affiliations:** Leibniz-Institut für Wissensmedien, 72076 Tuebingen, Germany; Arizona State University, UNITED STATES

## Abstract

Information exchange is a crucial process in groups, but to date, no one has systematically examined how a group member’s relationship with a group can undermine this process. The current research examined whether disidentified group members (i.e., members who have a negative relationship with their group) strategically undermine the group outcome in information exchange. Disidentification has been found to predict negative group-directed behaviour, but at the same time disidentified members run the risk of being punished or excluded from the group when displaying destructive behaviour. In three studies we expected and found that disidentified group members subtly act against the interest of the group by withholding important private information, while at the same time they keep up appearances by sharing important information that is already known by the other group members. These findings stress the importance of taking a group member’s relationship with a group into account when considering the process of information exchange.

## Introduction

Information exchange is a crucial process in groups [1–3]. Many important economic, technical, medical, or political decisions are made by groups [3,4]. Ideally, group members possess different pieces of important information that, if put together, increase the quality of any decision made by their group. Thus, the importance of open and honest information exchange where all group members share all the information they possess is obvious for achieving high quality decisions. But what if some group members *actively* undermine the process of information exchange? This could potentially have devastating results. The current research set out to investigate how a group member’s relationships with a group affects what type of information they share, withhold, falsify or destroy during information exchange.

Until recently, research has widely assumed that information exchange is cooperative [5], and has mostly focused on the underlying cognitive processes of information exchange [4]. More recent research, however, demonstrated that group members can be strategic with regards to what information they share with other group members [6–10]. Research, for example, found that group members consider information sharedness and information importance when they share their information [6]. Information sharedness refers to the amount of information that is known by all group members (public or private) and information importance refers to the importance to the problem at hand (important or unimportant). Information that is shared with other group members better predicts group judgements than information that group members keep to themselves, but if group members fail to share and heed uniquely held relevant information it can result in poorer decision quality [5,11,12]. Thus, to act in the interest of the group, it is important to share as much information as possible, especially important information that is unknown by the other group members. If a group member were to act *against* the interest of the group, there are, however, several possible ways to undermine the group performance. Most extreme forms of behaviour would be to destroy or even falsify all available information, but group members may also deliberately withhold information from their fellow group members. There is considerable research that has focused on the underlying motivations of why group members sometimes withhold (e.g., [6–8]) or distort (e.g., [6,13–15]) information in information exchange, but research that focusses on the relationship between a group member and a specific group as an underlying mechanism is lacking. In the current research we argue that a group member’s relationship with a group is an important, but hitherto neglected, factor that affects information exchange in group settings.

Considerable research has documented how the self is construed in relation to one’s group memberships [16,17]. When these relationships are positive, group members identify with the group; they perceive and describe themselves as a typical group member and they act and feel on behalf of the group membership [18–20]. When this relationship is negative, group members disidentify with this group; they perceive and describe themselves as different from other group members, they feel bad about the group membership, and they act against the interest of the group [21–24]. For identified group members, the group’s successes and failures are like their own, because the group membership is an important part of their self-concept [25]. As such, identified group members behave in a manner consistent with collective interests (even when it involves harming their own personal interests). They conform to group norms, because normative behaviour is perceived as being in the best interests of the group [2,18,26–28]. In support of this view, research found that social identification positively affects group members' contributions in a public goods dilemma [29]. Thus, it is expected that identified group members act in the interest of the group (i.e., share more information) in information exchange. Disidentified group members, on the other hand, demonstrate anti-normative behaviour [21,30,31], and even negative behaviour that harms the group interest directly [22,23]. Therefore, it is expected that disidentified group members would be motivated to undermine the process of information exchange.

Disidentified group members still see themselves as part of the group, thus they are at risk of being excluded [32,33] or punished [34–37] by the other group members if they act against the interest of the group. Recent research demonstrated the importance of a group member’s actions within a group in information exchange [32]. When group members deviate from group norms in information exchange (i.e., withhold or falsify information, instead of sharing information which is considered normative) they tend to get excluded by the other group members. This finding is in line with previous theory which suggests that punishment of norm violators is a key mechanism to promote cooperation in groups [34–37]. Although, disidentified group members have a negative relationship with their group, they still see themselves as part of the group, and are often not able to leave this group due to low permeability or because the benefits of staying in the group outweigh the costs of leaving the group [38]. Thus, even for disidentified group members it can be disagreeable or painful to be excluded and punished by the group, because in the end they are dependent on the group.

Moreover, reputation is a currency that is valid in many social settings [39–41]. According to this view, a group member’s reputation in information exchange within a group is established by his or her previous actions (sharing, withholding, distorting or destroy information). A group member is likely to be punished by the others group members in case that member has a bad reputation, and rewarded in case that member has a good reputation. Thus, behaviour of disidentified group members in information exchange may be driven by the desire to acquire a good reputation, while at the same time they subtly undermine the group outcome in information exchange. In order to do so, we suggest that disidentified group members strategically differentiate between private and public as well as important and unimportant information. Undermining the group subtly is possible by withholding information—which is the least extreme form of undermining the group outcome—from the other group members. This is especially harmful for the group if this information is important and unknown by the other group members. It is therefore expected that disidentified group members withhold more private important information than identified group members. In addition, we don’t expect any differences between the withholding of the other types of information for disidentified group members. Therefore, we hypothesize the following:

**Hypothesis 1a:** disidentified group members withhold more private important information than identified group members.
**Hypothesis 1b:** disidentified group members withhold more private important information compared to public important, public unimportant, and private unimportant information.

At the same time, however, disidentified group members need to keep up the appearance of cooperation (see also [7,13], for similar arguments). If disidentified group members withhold private important information, their destructive behaviour is easily detectable because they would also share less information in total. But sharing more important public information might be a strategy for disidentified group members to cover up their subversive behaviour, and thus reduce the risk of being punished or excluded from the group. Sharing public information has low costs, because it is already known by the other group members. As such, the other group members are already aware of the importance of the available public information. Thus, when a group member predominantly shares important public information it will help increase, or sustain the group member’s reputation, because the other group members can easily verify its importance. Therefore, we first, expect that disidentified group members might compensate their destructive behaviour by sharing more important public information compared to identified group members. Second, it is expected that disidentified group members share the largest amount of important public information because it is important but has low cost for the individual group member; the smallest amount of important private information because it is important but has the highest cost for the individual group member; public and private unimportant information will be shared at an intermediate level because this information is unimportant. Thus, we hypothesize the following:

**Hypothesis 2a:** disidentified group members share more important public information than identified group members.
**Hypothesis 2b:** the amount of information that disidentified group members share with the other group members differs in the following order ranging from high to low: public important, public unimportant, private unimportant, and private important.

To test whether information exchange is dependent on people’s relationships with their group, we conducted three studies in which we manipulated disidentification and social identification with an artificial group. After the manipulation, the participants took part in an information pooling game [6]. The information that could be exchanged was either public or private, and either important or unimportant. Participants had to decide which information they wanted to share, withhold, make less informative, distort, or destroy. One could argue that in case a group member refrains from sharing information with the other group members, that group member is, thus, withholding information. Although *not sharing* of information and *withholding* of information both are equivalent in terms of outcomes, they are not psychologically equivalent because they involve different decision frames (cf. [42]). In the first option the emphasis is on cooperating with the group, whereas in the second option the emphasis is on undermining the group. If group members decide not to share a piece of information, they most likely do not care about the group outcome. But withholding a piece of information is very deliberate and aims at harming the group. Thus, by focussing on both types of decisions—sharing and withholding of information—, it allows us to investigate how disidentified group members *deliberately* act against the interest of the group, and at the same time how they are keeping up the appearance of cooperation. In Studies 1 till 3 we tested whether disidentified group members withhold more important private information compared to identified group members and whether disidentified group members keep up the appearance of cooperation by sharing more public important information.

## Study 1

### Method

#### Ethics statement

The study was approved by the Ethics Committee of the Leibniz-Institut für Wissensmedien. Written informed consent was obtained from all participants.

#### Participants and design

A total of 56 students from the University of Tuebingen (38 female, median age 24 years, range 19–60 years) participated in a lab study in exchange for 4 euros. The current experiment took approximately 15 minutes to complete and was only a minor part of a study session for which the “main” study only required a small sample to test material. We aimed, however, to have at least 20 participants per cell. One participant who was familiar with the information pooling game was removed from the sample. Participants were randomly assigned to one of two conditions: disidentification condition (*N* = 31) and identification condition (*N* = 24).

#### Procedure

At the start of the experimental session, approximately six participants were seated in front of a computer, which displayed all instructions. The study was introduced as an investigation on decision making in a group context. Participants were informed that they would be randomly assigned to a group and that, prior to the experiment, the university is interested in their opinion on a current issue at the university. Hereupon, participants were told that in the actual experiment they would be working together in a computer game, set-up in a network, in which they could exchange information with other group members to find a hidden treasure.

After the general instructions and the group assignment, disidentification and social identification with the working group were manipulated. As disidentification and social identification are driven by feelings of coherence versus contradiction to the group [22,23,43–45], the relationship with the group was manipulated using a similarity versus difference manipulation on a relevant topic. Participants were informed that the university is proposing to introduce a “veggie day” in the university cafeteria and that the university is collecting the opinion of several students on this matter. Participants gave their opinion about vegetarian food and meat consumption and were asked whether they are in favour of or against a “veggie day” in the university cafeteria. After completing these questions, participants received a summary of their opinion on vegetarian food, and a bogus summary of the other group members. In the *disidentification condition*, the opinion from the participant was opposite to that of the other group members, whereas in the *identification condition* the opinion from the participant was similar to that of the other group members.

After the manipulation, the participants completed measures of disidentification and identification with the assigned group as manipulation checks, and then continued to the experimental task in which participants had to exchange 12 pieces of information (cues) in order to find a hidden treasure buried in a grave in the graveyard of a monastery [6]. All participants were told that each group member was given 12 pieces of information which they could exchange with the other group members. To be able to find the treasure it is important to pool as many cues as possible. Similar to the original paradigm, the information was either public or private, and either important (i.e., excluded 64 graves) or unimportant (i.e., excluded 4 graves).The task was transparent in that participants knew the importance and sharedness of each piece of information. This explicit information is important because it allows participants to be strategic in information sharing and withholding (see [7], for a similar argument). Participants had to decide which information they wanted to share, withhold, or distort. In addition, in this experiment participants were given a fourth option, in which they could destroy the information, making it no longer accessible for the participant and the other group members. Given that the participants were given additional options, the test for our hypotheses may be considered more conservative compared to the original paradigm. All participants were told that in order to find the treasure it is important that they share as much information as possible with the other group members. No additional reward was offered if the treasure was found. After the information exchange, participants were debriefed and thanked.

#### Measures

In Study 1 through 3, all ratings were made on 7-point scales ranging from 1 (*not at all*) to 7 (*very much*) unless indicated otherwise. *Disidentification* with the group was measured with 11 items (α = .91, *M* = 2.75, *SD* = 1.35; e.g., ‘I wish I had nothing to do with this group’) adapted from Becker and Tausch [22]. *Social identification* with the group of was measured with 3 items (α = .94, *M* = 2.79, *SD* = 1.49; e.g., ‘I identify with this group’) adapted from Doosje, Ellemers, and Spears [46]. *Information exchange* was coded by counting how many cues from each of the four categories were shared, withheld, distorted, and destroyed. Each score (the number of important public, important private, unimportant public, and unimportant private pieces of information) could range from zero to three.

### Results

#### Manipulation check

A multivariate analysis of variance (MANOVA) with condition as a between-subject factor and disidentification and social identification as dependent variables was conducted. Results revealed a significant main effect of condition, *F*(2, 22) = 17.21, *p* < .001, η^2^_p_ = .40. As expected, participants in the disidentification condition disidentified more (*M* = 3.42, *SD* = 1.30), compared to the identification condition (*M* = 1.89, *SD* = 0.85), *F*(1, 53) = 25.44, *p* < .001, η^2^_p_ = .32. Participants in the identification condition identified more (*M* = 3.64, *SD* = 1.68), compared to the disidentification condition (*M* = 2.13, *SD* = 0.88), *F*(1, 53) = 18.50, *p* < .001, η^2^_p_ = .26.

#### Information exchange

To test our two hypotheses, we first conducted two separate ANOVA’s for the number of shared public important information and withheld private important information with condition (disidentification and identification) as a between-subjects variable. In line with Hypothesis 1a, we found that participants in the disidentification condition withheld more important private information (*M* = 1.32, *SD* = 0.94), compared to participants in the identification condition (*M* = 0.75, *SD* = 1.07), *F*(1, 53) = 4.41, *p* < .05, η^2^_p_ = .08. Unexpectedly, we did not find support for Hypothesis 2a, that participants in the disidentification condition shared more important public information (*M* = 1.90, *SD* = 1.16), compared to participants in the identification condition (*M* = 2.25, *SD* = 0.99), *F*(1, 53) = 1.36, *p* = .25, η^2^_p_ = .03.

**Fig 1:**
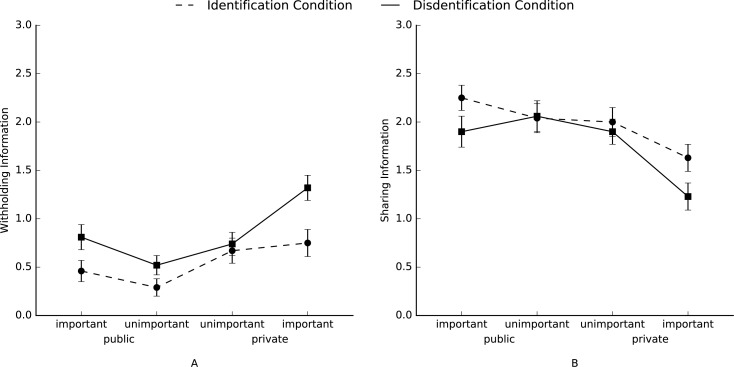
**Mean levels of each type of information which participants (A) withheld, or (B) shared in Study 1.** Error bars represent ±1 SE of the mean.

To test whether disidentified group members strategically differentiate between private and public as well as important and unimportant information we conducted separate repeated-measures analysis of variance (RM-ANOVA) with *a priori* contrasts for the number of shared, withheld, distorted, and deleted information, with condition (disidentification and identification) as a between-subjects variable, and with sharedness (public vs. private) and importance (important vs. unimportant) as a within-subjects variable. To test Hypothesis 1b, we used planned contrasts which predicted that disidentified group members (versus identified group members) would withhold more private important information compared to the other types of information (contrast coefficients: –3, –1, –1, –1). Although, the planned contrast which compared both conditions was not significant, *F*(1, 53) = 1.41, *p* = .24, η^2^_p_ = .03 (Fig 1A), it did reveal a significant simple effect for disidentification, *F*(1, 53) = 10.19, *p* < .01, η^2^_p_ = .16. The simple effect for identification was not significant, *F*(1, 53) = 1.51, *p* = .22, η^2^_p_ = .03. Thus, participants in the disidentification condition withheld more important private information compared to the other types of information; there were no difference between the different types of information for participants in the identification condition. To test Hypothesis 2b, we used planned contrasts in which we tested whether the amount of information that disidentified group members (versus identified group members) would share with the other group members differs in the following order ranging from high to low: public important, public unimportant, private unimportant, and private important (contrast coefficients: 3, 1, –1, –3, respectively). Although, our planned contrast which compared both conditions was not significant, *F*(1, 53) < 1 (Fig 1B), it did reveal a significant simple effect for disidentification, *F*(1, 53) = 9.90, *p* < .01, η^2^_p_ = .16, and for identification, *F*(1, 53) = 5.85, *p* < .05, η^2^_p_ = .10. Thus, both participants in the identification and disidentification condition shared the most important public information and the least important private information. Finally, there were no main or interaction effects of condition for distorting information, or deleting information, *F*s < 1.83, *p* > .18.

### Discussion

In Study 1, we found evidence that participants in the disidentification condition withhold more important private information compared to participants in the identification condition. Moreover, we also found that participants in the disidentified withhold more important private information in comparison to the other types of information. There were no differences between the different types of information that participants in the identification condition withheld from the other group members. Thus, disidentified group members appear to be strategic in what type of information they withhold from the other group members.

The results from Study 1 revealed that participants in the disidentification condition shared the largest amount of important public information and the smallest amount of important private information. But the same pattern also appeared for participants in the identification condition. Moreover, we did not find support that the participants in the disidentification condition share more important public information compared to participants in the identification condition.

Although, we found some support for our hypotheses, the evidence is still limited. This could be due to low statistical power—the expected interaction effects did not reach the conventional levels of significance—or due to the presentation of each piece of information available in the information exchange. The available information in Study 1 was displayed in one overview, which might cause participants to be more balanced in their decision. When information is presented sequentially it makes the prior decision salient, thereby increases commitment to that decision [47]. Thus, we expect group members to be more strategic in their choices when the information is presented sequentially, compared to a more balanced strategy when all information is displayed in one overview.

The findings from Study 1 are somewhat similar to earlier research on the effects of pro-social versus pro-self motivations [6]. One could argue that the information exchange in the present data was motivated by a pro-social (i.e., the goal to cooperate to maximise outcomes for both oneself and the group) versus a pro-self (i.e., the goal it to maximise one’s own outcomes relative to the group) orientation [48]. Participants worked on a group task in which they had to pool as much information as possible in order to succeed as a group. There were no other incentives available other than group success, which renders the option of free-riding, that is, to profit from the contributions of others without making a contribution themselves highly improbable. Even if our participants were primarily concerned with the welfare of themselves, it would still be in their personal interest to succeed as a group—e.g., basking in reflected glory [25]. Thus, if participants in the disidentification condition were motivated by a pro-self orientation one would expect behaviour that maximizes the personal gain in the situation, which would be to succeed as a group. Our findings from Study 1, on the other hand, demonstrated that disidentified group members strategically acted *against* the interest of the group, leaving them with zero outcomes, except for group failure. Therefore, it is reasonable to assume that pro-self orientation and disidentification are indeed distinct processes. Thus, the main objective of Study 2 was to examine if our finding still occur if we control for pro-social versus pro-self motivations, which will provide support for our notion that a group member’s relationship with a group is an important factor that affects information exchange in group settings. In Study 2 we also aimed at increasing the sample size, and thus statistical power. Moreover, the information in the information paradigm will be presented sequentially.

## Study 2

### Method

#### Ethics statement

The study was approved by the Ethics Committee of the Leibniz-Institut für Wissensmedien. Written informed consent was obtained from all participants.

#### Participants and design

A total of 99 students from the University of Tuebingen (78 female, median age 22 years, range 18–30 years) participated in a lab study in exchange for 4 euros. The experiment took approximately 15 min to complete and was part of a longer session that included another unrelated study. Data collection was scheduled for a specific time frame in which we aimed to recruit as many participants as possible (but at least 30 per cell). Two participants who were removed from the sample because they were the only participant attending at a particular session—i.e., there was no group available. Participants were randomly assigned to one of two conditions: disidentification condition (*N* = 44) and identification condition (*N* = 53).

#### Procedure

The procedure and measures were identical to Study 1, except that the participants received the pieces of information one after the other (i.e., sequential). In this study, participants were also given a fifth option, namely to make the information less informative. To give an example, one of the cues in the paradigm stated “the monk did not die in October”. Making this item less informative, would change this piece of information to “the monk did not die in autumn”. As such, this is a less extreme form of distorting information, because the cue is still correct. Given that the participants had an additional option, the test for our hypotheses may be considered as more conservative compared to Study 1. In addition, we measured participant’s pro-social and pro-self motivations.

#### Measures

We used the same measures as in Study 1 for *disidentification* (α = .92, *M* = 2.50, *SD* = 1.14), and *social identification* (α = .87, *M* = 3.31, *SD* = 1.44). Moreover, the same coding schema for *information exchange* was used as in Study 1. *Pro-social* motivation was measured with two items (α = .78, *M* = 4.85, *SD* = 1.65; e.g., ‘In the treasure hunt I wanted someone in my group to find the treasure’). *Pro-self* motivations were also assessed with two items (α = .72, *M* = 4.94, *SD* = 1.57; e.g., ‘In the treasure hunt I wanted to find the treasure myself’). Both pro-social and pro-self motivations were negatively correlated with each other (*r*(97) = -.36, *p* < .001)

### Results and discussion

#### Manipulation check

A MANOVA with condition as a between-subject factor and disidentification and social identification as dependent variables was conducted. Results revealed a significant main effect of condition, *F*(2, 94) = 19.61, *p* < .001, η^2^_p_ = .29. As expected, participants in the disidentification condition disidentified more (*M* = 3.12, *SD* = 1.11), compared to the identification condition (*M* = 1.98, *SD* = 0.89), *F*(1, 95) = 31.80, *p* < .001, η^2^_p_ = .25. Participants in the identification condition identified more (*M* = 3.92, *SD* = 1.54), compared to the disidentification condition (*M* = 2.58, *SD* = 0.88), *F*(1, 95) = 26.63, *p* < .001, η^2^_p_ = .22.

#### Information exchange

As in Study 1, we first conducted two separate ANOVA’s for the number of shared public important information and withheld private important information with condition (disidentification and identification) as a between-subjects variable. In line with Hypothesis 1a, we found that participants in the disidentification condition withheld more important private information (*M* = 0.77, *SD* = 0.86), compared to participants in the identification condition (*M* = 0.45, *SD* = 0.70), *F*(1, 95) = 4.11, *p* < .05, η^2^_p_ = .04. Unexpectedly, but in line with Study 1, we did not find support for Hypothesis 2a, and participants in the disidentification condition did not share more important public information (*M* = 1.73, *SD* = 1.00), compared to participants in the identification condition (*M* = 1.70, *SD* = 1.10), *F*(1, 95) < 1.

**Fig 2:**
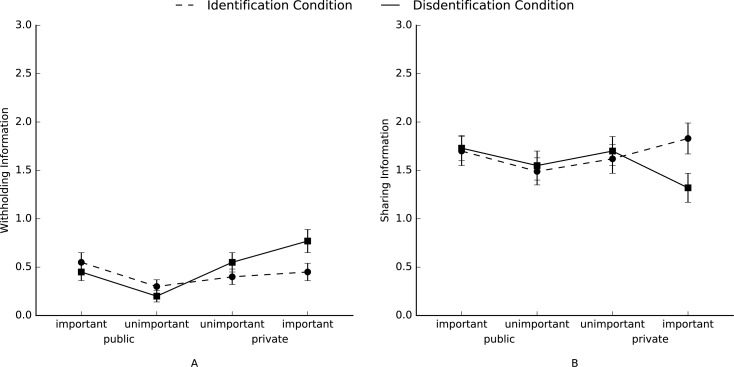
**Mean levels of each type of information which participants (A) withheld, or (B) shared in Study 2.** Error bars represent ±1 SE of the mean.

Again, to test whether disidentified group members strategically differentiate between private and public as well as important and unimportant information we conducted separate RM-ANOVA’s with *a priori* contrasts for the number of shared, withheld, making information less informative, distorted, and deleted information, with condition (disidentification and identification) as a between-subjects variable, and with sharedness (public vs. private) and importance (important vs. unimportant) as a within-subjects variable. A planned contrast (Hypothesis 1b) which predicted that disidentified group members (versus identified group members) would withhold more private important information compared to the other types of information (contrast coefficients: –3, –1, –1, –1), was significant, *F*(1, 95) = 4.69, *p* < .05, η^2^_p_ = .05 (Fig 2A). Simple effects revealed that this effect was significant for the disidentification condition, *F*(1, 95) = 10.63, *p* < .01, η^2^_p_ = .10, but not for the identification condition, *F*(1, 95) < 1. Thus, as in Study 1, participants in the disidentification condition withheld more important private information compared to the other types of information; there were no difference between the different types of information for participants in the identification condition. A planned contrast (Hypothesis 2b) which predicted that the amount of information that disidentified group members (versus identified group members) would share with the other group members differs in the following order ranging from high to low: public important, public unimportant, private unimportant, and private important (contrast coefficients: 3, 1, –1, –3, respectively), was also significant, *F*(1, 95) = 5.39, *p* < .05, η^2^_p_ = .05 (Fig 2B). Simple effects revealed that this effect was significant for the disidentification condition, *F*(1, 95) = 4.42, *p* < .05, η^2^_p_ = .04, but not for the identification condition, *F*(1, 95) = 1.30, *p* < .26, η^2^_p_ = .01. Thus, as in Study 1, participants in the disidentification condition shared the most important public information and the least important private information; this pattern was not significant for participants in the identification condition. Finally, there were no main or interaction effects of condition for making information less informative, distorting information, or deleting information, *F*s < 2.25, *p* > .13.

#### Pro-social versus pro-self motivations

In order to test whether pro-social and pro-self motivations were affected by our manipulation we conducted a MANOVA with condition (disidentification and identification) as a between-subjects variable. We found, as expected, that pro-social and pro-self motivations did not differ between conditions, *F*s < 1.37, *p* > .24. Next, we conducted two hierarchical multiple regression analyses where we included pro-social and pro-self motivations into the regression analysis as the first predictor, followed by condition. We found that in the first step, pro-social motivations (*B* = -0.20, *SE* = 0.05, 95% CI = (-0.29, -0.11), *p* < .001) decreased the amount of important private information participants withheld from the other group members, but pro-self motivations did not (*B* = 0.03, *SE* = 0.05, 95% CI = (-0.07, 0.12), *p* = .56), *F*(2, 96) = 12.18, *p* < .001, *R*^2^ = .20. When we included condition in the next step, pro-social motivations remained significant, but condition was, as expected, also a significant predictor (*B* = 0.32, *SE* = 0.14, 95% CI = (0.04, 0.60), *p* < .05), *F*(3, 95) = 10.18, *p* < .001, Δ*R*^2^ = .04. Although, as reported above, we did not find that participants in the disidentification condition share more important public information, compared to participants in the identification condition, we did find that pro-social motivations increased (*B* = 0.22, *SE* = 0.06, 95% CI = (0.10, 0.35), *p* < .001) and pro-self motivations decreased (*B* = -0.14, *SE* = 0.06, 95% CI = (-0.26, -0.01), *p* < .05) the amount of important public information participants share with the other group members, *F*(2, 96) = 11.90, *p* < .001, *R*^2^ = .22.

### Discussion

The results revealed that participants in the disidentification condition withhold more important private information compared to participants in the identification condition. Moreover, as in Study 1, we found that participants in the disidentified withhold more important private information in comparison to the other types of information. There were no differences between the different types of information that participants in the identification condition withheld from the other group members. Thus, disidentified group members appear to be strategic in what type of information they withhold from the other group members.

Similar as in Study 1, we did not find support that the participants in the disidentification condition share more important public information compared to participants in the identification condition. However, we did find that participants in the disidentification condition are strategic in what type of information they share with the other group members. Similar as in Study 1 we found that participants in the disidentification condition shared the largest amount of important public information, and the smallest amount of important private information. Moreover, there were no differences between each type of information which was shared by participants in the identification condition.

We also found support that the amount of important private information which participants withheld from the other group members could not only be attributed to a pro-self or pro-social motivation, but our manipulation had an additional effect. The amount of important public information participants share with the other group members was only affected by pro-self and pro-social motivations. This is in line with earlier work, that pro-self and pro-social motivations affect the amount of information people share with the other group members [7,49].

Although, the results showed that participants in the disidentification condition are strategic in what type of information they share with the other group members—i.e., they share the most important public information and the least important private information—, we were not able to find support that the participants in the disidentification condition share more important public information compared to participants in the identification condition. It appears that participants in the disidentification condition share equal amounts of this type information. However, this evidence, of course is limited. To provide more compelling evidence, we decided to measure behavioural intentions regarding information exchange (i.e., if they would rather use a more extreme form of behaviour) if they were not dependent on the information of the other group members. Behavioural intentions capture hypothetical behaviour that group members would like to display if they did *not* have to keep up appearances. We would expect that behavioural intentions regarding information exchange would be more extreme (e.g. destroy or falsify information) than actual behaviour, but only for participants in the disidentification condition. Moreover, we expect that differences between behavioural intentions and actual behaviour are largest for the kind of information where destructive behaviour cannot be easily disguised. Thus, we expect a similar trend for differences between behavioural intentions and actual behaviour as we predicted for sharing of information. Thus, for participants in the disidentification condition we expect the difference between behavioural intentions and actual behaviour to be the largest (i.e., they would rather use a more extreme form of behaviour) for public important information, and to be the smallest for important private information (i.e., where their destructive behaviour will not be noticed by other group members but has a great impact on the group outcome); we expect differences between behavioural intentions and actual behaviour to be at an intermediate level for public and private unimportant information. For participants in the identification condition we don’t expect any differences between behavioural intentions and actual behaviour for each type of information.

## Study 3

### Method

#### Ethics statement

The study was approved by the Ethics Committee of the Leibniz-Institut für Wissensmedien. Written informed consent was obtained from all participants.

#### Participants and design

A total of 75 students from the University of Tuebingen (59 female, median age 23 years, range 18–51 years) participated in a lab study in exchange for 4 euros. The experiment took approximately 15 min to complete and was part of a longer session that included a number of different studies. Data collection was scheduled for a specific time frame in which we aimed to recruit as many participants as possible (but at least 30 per cell). Participants were randomly assigned to one of two conditions: disidentification condition (*N* = 33) and identification condition (*N* = 42).

#### Procedure

The procedure and measures were identical to Study 2, except that we did not assess pro-self and pro-social motivations in the current study. In addition, we measured participant’s intentions regarding information exchange.

#### Measures

We used the same measures for *disidentification* (α = .91, *M* = 2.27, *SD* = 0.99), and *social identification* (α = .88, *M* = 3.68, *SD* = 1.51) as in Study 1 and Study 2. Moreover, the same coding schema for *information exchange* was used as in both studies. *Intentions* were assessed by asking participants after each information item what they would rather do (1 = share, 2 = withhold), 3 = make less informative, 4 = distort, or 5 = destroy) if they were not dependent on the information of the other group members. Extremity of intention for each item was calculated by subtracting the intention score from the information exchange score, so that larger values indicate more extreme intentions, whereas scores of zero indicate that intentions do not deviate from information exchange.

### Results and discussion

#### Manipulation check

A MANOVA with condition as a between-subject factor and disidentification and social identification as dependent variables was conducted. Results revealed a significant main effect of condition, *F*(2, 72) = 26.10, *p* < .001, η^2^_p_ = .42. As expected, participants in the disidentification condition disidentified more (*M* = 2.90, *SD* = 0.98), compared to the identification condition (*M* = 1.77, *SD* = 0.66), *F*(1, 73) = 38.29, *p* < .001, η^2^_p_ = .33. Participants in the identification condition identified more (*M* = 4.39, *SD* = 1.39), compared to the disidentification condition (*M* = 2.78, *SD* = 1.13), *F*(1, 73) = 29.20, *p* < .001, η^2^_p_ = .29.

#### Information exchange

As in Study 1, we first conducted two separate ANOVA’s for the number of shared public important information and withheld private important information with condition (disidentification and identification) as a between-subjects variable. In line with Hypothesis 1a, we found that participants in the disidentification condition withheld more important private information (*M* = 1.03, *SD* = 0.88), compared to participants in the identification condition (*M* = 0.38, *SD* = 0.66), *F*(1, 73) = 13.27, *p* < .001, η^2^_p_ = .15. We also found support for Hypothesis 2a, that participants in the disidentification condition shared more important public information (*M* = 1.97, *SD* = 0.92), compared to participants in the identification condition (*M* = 1.40, *SD* = 0.92), *F*(1, 73) = 6.42, *p* < .05, η^2^_p_ = .08.

**Fig 3:**
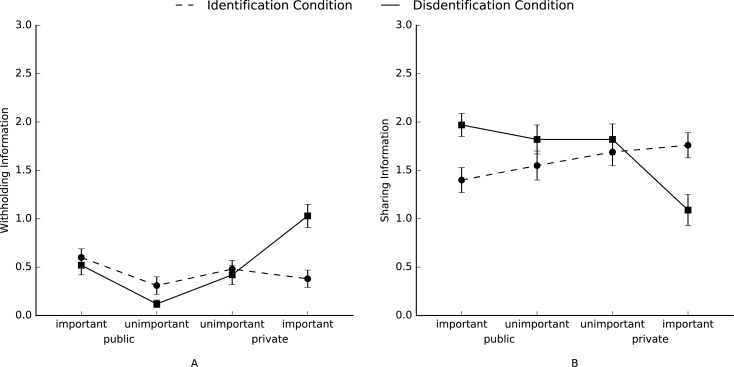
**Mean levels of each type of information which participants (A) withheld, or (B) shared in Study 3.** Error bars represent ±1 SE of the mean.

Again, to test whether disidentified group members strategically differentiate between private and public as well as important and unimportant information we conducted separate RM-ANOVA’s with *a priori* contrasts for the number of shared, withheld, making information less informative, distorted, and deleted information, with condition (disidentification and identification) as a between-subjects variable, and with sharedness (public vs. private) and importance (important vs. unimportant) as a within-subjects variable. A planned contrast (Hypothesis 1b) which predicted that disidentified group members (versus identified group members) would withhold more private important information compared to the other types of information (contrast coefficients: –3, –1, –1, –1), was significant, *F*(1, 73) = 17.68, *p* < .001, η^2^_p_ = .20 (Fig 3A). Simple effects revealed that this effect was significant for the disidentification condition, *F*(1, 73) = 17.68, *p* < .001, η^2^_p_ = .26, but not for the identification condition, *F*(1, 73) < 1. Thus, as in Study 1 and Study 2, participants in the disidentification condition withheld more important private information compared to the other types of information; there were no difference between the different types of information for participants in the identification condition. A planned contrast (Hypothesis 2b) which predicted that the amount of information that disidentified group members (versus identified group members) would share with the other group members differs in the following order ranging from high to low: public important, public unimportant, private unimportant, and private important (contrast coefficients: 3, 1, –1, –3, respectively), was also significant, *F*(1, 73) = 21.04, *p* < .001, η^2^_p_ = .22 (Fig 3B). Simple effects revealed that this effect was both significant for the disidentification condition, *F*(1, 73) = 17.61, *p* < .001, η^2^_p_ = .19, and for the identification condition, albeit in the opposite direction, *F*(1, 73) = 4.76, *p* < .05, η^2^_p_ = .06. Thus, participants in the disidentification condition shared the most important public information and the least important private information, whereas participants in the identification condition shared the most important private information and the least important public information. Finally, there were again no main or interaction effects of condition for making information less informative, distorting information, or deleting information, *F*s < 1.61, *p* > .20.

#### Behavioural intentions

It was expected that participants in the disidentification would rather use more extreme forms of behaviour compared to participants in the identification condition, but only for important public information. When analysing participant’s intentions we indeed found that compared to participants in the identification condition (*M* = 0.03, *SD* = 0.61), participants in the disidentification condition would rather use a more extreme form of behaviour (e.g., destroy or falsify), but only for important public information (*M* = 0.36, *SD* = 0.66), *F*(1, 73) = 5.10, *p* < .05, η^2^_p_ = .07; other *F*s < 2.29, *p* > 13. Thus, the behavioural intentions regarding important public information for participants in the disidentification condition were more extreme (e.g., destroy or falsify information) than actual behaviour—i.e., they were keeping up appearances.

To investigate the underlying pattern of differences between behavioural intentions and actual behaviour we conducted a RM-ANOVA with condition (disidentification and identification) as a between-subjects variable, and with sharedness (public vs. private) and importance (important vs. unimportant) as a within-subjects variable. A planned contrast which predicted that for each type of information the difference between behavioural intentions and actual behaviour for disidentified group members (versus identified group members) differs in the following order ranging from high to low: public important, public unimportant, private unimportant, and private important (contrast coefficients: 3, 1, –1, –3, respectively), was significant, *F*(1, 73) = 5.92, *p* < .05, η^2^_p_ = .08. Simple effects revealed that this effect was only significant for the disidentification condition (*M* = 0.36, *SD* = 0.66, *M* = 0.28, *SD* = 0.85, *M* = 0.22, *SD* = 0.77, and *M* = -0.03, *SD* = 0.81), *F*(1, 73) = 6.99, *p* = .01, η^2^_p_ = .09, and not for the identification condition (*M* = 0.03, *SD* = 0.61, *M* = 0.12, *SD* = 0.85, *M* = 0.00, *SD* = 0.49, and *M* = 0.17, *SD* = 0.56), *F*(1, 73) < 1. Thus, for participants in the disidentification condition the difference between behavioural intentions and actual behaviour was the largest (i.e., they would rather use a more extreme form of behaviour) for public important information, whereas there was only a little difference between behavioural intentions and actual behaviour for important private information. For participants in the identification condition the differences between behavioural intentions and actual behaviour did not differ for each type of information.

### Discussion

Results demonstrated that participants in the disidentification condition withhold more important private information compared to participants in the identification condition. Moreover, participants in the disidentified withhold more important private information in comparison to the other types of information. There were no differences between the different types of information that participants in the identification condition withheld from the other group members.

In Study 3, we also found first evidence that participants in the disidentification condition withhold more important public information compared to participants in the identification condition. Moreover, we again found that participants in the disidentification condition shared the largest amount of important public information, and the smallest amount of important private information. The opposite effect occurred for participants in the identification condition—i.e., they shared the most amount of important private information and the least amount of important public information. In addition, we found that participants in the disidentification preferred to use more extreme forms of destructive behaviour (e.g., distort or destroy information) when it comes to important public information compared to participants in the identification condition, but instead kept up appearances by sharing useless information.

## Meta-analysis

To deal with any issues created by a lack of statistical power in the individual studies [50,51], and to test the robustness of the effects, we conducted several meta-analyses using fixed effects in which the mean effect size (i.e., mean correlation) was weighted by sample size. Given the sample sizes in our three studies, the power (two-tailed) to detect a medium effect size of 0.29 (*d* = 0.60) in the meta-analyses is 0.993. The effects for Hypothesis 1a and 1b were not heterogeneous, *Q*s < 4.39, *p* > .11, indicating that a fixed-effects analysis was appropriate. As predicted, participants in the disidentification condition withheld more important private information, compared to participants in the identification condition, *r* = -.31, *SE*_*r*_ = 0.06, 95% CI = (-0.43, -0.19), *z* = -5.14, *p* < .001. Moreover, our predicted contrast that disidentified group members (versus identified group members) would withhold more private important information compared to the other types of information, was also significant, *r* = -.33, *SE*_*r*_ = 0.06, 95% CI = (-0.44, -0.21), *z* = -5.60, *p* < .001. Simple effects revealed that this effect was only significant for the disidentification condition, *r* = .43, *SE*_*r*_ = 0.05, 95% CI = (0.33, 0.54), *z* = 8.04, *p* < .001, but not for the identification condition, *r* = .02, *SE*_*r*_ = 0.07, 95% CI = (-0.11, 0.15), *z* = 0.32, *p* = .75.

The effects for Hypothesis 2a and 2b were heterogeneous, *Q*s < 13.25, *p* > .001, so we decided to conduct both fixed-effects and random-effects analyses. The results of both analyses are similar, so we only report the results of the fixed-effects analyses. Contrary to Hypothesis 2a, we did not find robust evidence that participants in the disidentification condition share more important public information, compared to participants in the identification condition, *r* = -.09, *SE*_*r*_ = 0.06, 95% CI = (-0.22, 0.03), *z* = -1.42, *p* = .16. However, our predicted contrast that the amount of information that disidentified group members (versus identified group members) would share with the other group members differs in the following order ranging from high to low: public important, public unimportant, private unimportant, and private important was significant, *r* = -.32, *SE*_*r*_ = 0.06, 95% CI = (-0.43, -0.21), *z* = -5.49, *p* < .001. Simple effects revealed that this effect was only significant for the disidentification condition, *r* = .36, *SE*_*r*_ = 0.06, 95% CI = (0.25, 0.48), *z* = 6.35, *p* < .001, but not for the identification condition, *r* = -.05, *SE*_*r*_ = 0.06, 95% CI = (-0.18, 0.07), *z* = -0.83, *p* = .41.

## General discussion

In three studies, we investigated whether disidentified group members undermine the group outcome in information exchange while keeping up the appearance of cooperation. It was expected and found that group members can be strategic with regards to what type of information they exchange with other group members and that this depends on a group member’s relationship with a group. More specifically, it was expected that disidentified (compared to identified) group members withhold more private important information but share more important public information that is already known by other group members. In three studies we found, as expected, that disidentified group members withhold more important private information than identified group members. Moreover, we found that disidentified group members shared the largest amount of important public information, and the smallest amount of important private information. Thus, disidentified group members appear to be strategic in what type of information they withhold from the other group members. In addition, we also found that withholding of information cannot exclusively be explained by pro-self and pro-social motivations; a group member’s relationship with a group was an additional factor. This finding provides further support for our notion that a group member’s relationship with a group is an important factor that affects information exchange in group settings.

Although, disidentified group members do not necessarily share more public important information than identified group members (only Study 3 supported this notion), they do share at least equal amounts of this type of information. More importantly, we found robust evidence that disidentified group members are strategic in what type of information they share with the other group members; the type of information disidentified group members shared the most was information which is already known by the other group members. In addition, in Study 3 we found that disidentified group members preferred to use more extreme forms of destructive behaviour (e.g., distort or destroy information) when it comes to important public information, but instead they acted pro-social towards the other group members. Thus, taken together, we do have support for our notion that disidentified group members are keeping up the appearance of cooperation.

The current findings contribute to a better understanding of destructive and negative forms of information exchange. They have important implications for research on group decision making [1] and social dilemmas [52]. Our results show that choices people make in information exchange go beyond a dichotomy of cooperation or defection. It was found that identified and disidentified group members are very strategic in what type of information (sharedness and importance) they share and withhold (see also [6]). As such, it is the type of information that can be exchanged and the type of behaviour that is chosen, which makes the information exchange more or less cooperative versus defective. Disidentified group members share more important public information, but simultaneously withhold important private information. At first glance, one could argue that this behaviour is cooperative, because more information is shared. However, it is important private information that holds the greatest value [5,11,12]. Therefore, by withholding this type of information one is putting the group at a greater disadvantage, and as such making its decisions defective. Thus, in order to fully understand both the positive and negative aspects of information exchange, it is necessary for future research to consider different types of information (sharedness and importance), as well as different types of constructive and destructive behaviour (e.g., share, withhold).

The data underline that disidentified group members find means and measures to undermine the group’s outcome. Disidentified group members do not, however, use extreme types of destructive behaviour in information exchange in order to harm the group openly (i.e., distorting and destroying information), but find subtle ways to undermine the group’s performance. Thus, it appears that the risk of being excluded from the group, or having a bad reputation, reduces the likelihood to display open hostile behaviour in information exchange. Even for disidentified group members, the benefit of remaining part of the group often outweighs the cost of getting punished or excluded from the group. However, disidentified members do have the tendency to act non-normatively and harm the group [22,23,30]. This premise is further supported by our finding that disidentified group members would have rather used more extreme types of behaviour if they were not dependent on the group. In order to solve this balancing act, disidentified group members pretended to be cooperative by sharing important information which is known by the other group members, while at the same time they kept important private information. Thus, the current data demonstrates that the inclusion of the members’ relationships to their group allows a better understanding of positive and negative behaviour in information exchange, and it is worthwhile to include both social identification and disidentification in future research.

Although this research is the first to provide evidence that disidentified group members strategically undermine the group outcome in information exchange, there are some limitations. We employed a modified minimal group paradigm in which participants were assigned to an artificial group. One could also argue that an artificial group is only short-term and therefore irrelevant for the self. However, this paradigm, although it does not involve existing group membership, is widely recognized as a valid way to induce the sense of group membership experimentally [53–55]. As such, it is a useful way to examine the causal role of social identification and disidentification in information exchange. It would, however, be interesting to see if our findings would also occur in information exchange in existing groups.

The participants in our research did not interact with one another, but it would be interesting to investigate how information exchange unfolds if group members do interact. Interaction allows the disidentified group members to get caught in the process of undermining the group outcome, which might force them to abandon and change their current strategy. Interaction can take place repeatedly for several iterations, or only once. Research has shown that punishments—or rewards—in iterated interactions are more likely to get norm-violators back on track compared to single interactions [13]. Thus, information exchange in an iterative fashion might help getting disidentified group members back on track again, but only if they get caught and punished for their subverting behaviour. This would also give us more insights into the threshold of how much subverting behaviour is allowed, before group members get punished or excluded by the other group members.

The present research offers practical implications for organizations. The data demonstrates that having a disidentified group member in your group can have detrimental outcomes because subversive behaviour might go unnoticed for a long time, if it is noticed at all. Considering that disidentified group members can have detrimental outcomes for information exchange—which is a common and important activity for organizations [3–4]—it is important that organizations are attentive to the development of disidentification for their employees and aware of the danger these team members may have for the group outcomes. Disidentified group members could, for instance, be asked directly to provide unique information. Even better still would be to take active measures to reduce disidentification of its employees. One way would be to would be to create a psychological fit between the organization and the employee so that employees are able to act in a way that “feels right” [38]. Unfortunately, to date, little research has focused on the prevention and reduction of disidentification, thus more research in this direction is needed.

Taken together, these studies demonstrate that disidentified members strategically act against the interest of the group, and in doing so they put on a convincing charade to keep the other group members from finding out. It appears that choices in information exchange go beyond the dichotomy of cooperation or defection, and that the type of information which can be exchanged and the type of behaviour are important elements to integrate into research in order to increase our understanding of all forms of information exchange processes. More importantly, these findings stress the importance of taking a group member’s relationships with a group into account when it comes to information exchange. It can hardly be in the interest of the group to have a member that undermines the process of information exchange.

## Supporting information

S1 File Data for the main analyses for Study 1. (SAV)

S2 File Data for the main analyses for Study 2. (SAV)

S3 File Data for the main analyses for Study 3. (SAV)
